# Experimental Investigation on Ablation of 4H-SiC by Infrared Femtosecond Laser

**DOI:** 10.3390/mi13081291

**Published:** 2022-08-11

**Authors:** Lukang Wang, You Zhao, Yu Yang, Manman Zhang, Yulong Zhao

**Affiliations:** State Key Laboratory for Manufacturing Systems Engineering, Xi’an Jiaotong University, Xi’an 710049, China

**Keywords:** infrared, femtosecond laser, silicon carbide, ablation threshold

## Abstract

Femtosecond laser ablation has become one of the important structural processing methods for the third-generation semiconductor material, silicon carbide (SiC), and it is gradually being employed in the manufacture of microelectromechanical systems and microelectronic devices. Experimental study has been performed on infrared single and multiple pulses (1035 nm) femtosecond laser ablation of SiC at various processing parameters. Diameters of laser ablation spots on 4H-SiC were measured to estimate the absorption threshold for material modification and structural transformation, which were 2.35 J/cm^2^ and 4.97 J/cm^2^, respectively. In the multiple-pulse scribing ablation for microgrooves, the ablation threshold dropped to 0.70 J/cm^2^ due to the accumulation effect when the effective pulse number reached 720. The calculated average of the thermally stimulated ablation depth of 4H-SiC is 22.4 nm, which gradually decreased with the raising of the effective pulse number. For obtaining square trenches with precise and controllable depths and a smooth bottom in 4H-SiC, the effects of processing parameters on the material removal rate and surface roughness are discussed. The ablation rate per pulse is almost constant, even if the effective pulse number varies. The reduction of laser spot overlapping ratio in x direction has a greater weakening effect on the material removal rate than that in y direction. The precise amount of material removal can still be controlled, while modulating the surface roughness of the ablated features by changing the hatch rotation angle. This research will help to achieve controllable, accurate, and high-quality machining results in SiC ablation, using infrared femtosecond laser.

## 1. Introduction

The prosperity of micro-electronics has been accompanied by the rapid rise of microelectromechanical systems (MEMS), of which silicon (Si) is currently the dominant material. Today, there is a growing requirement for devices that have the capacity to operate in harsh environments, especially high temperatures, beyond the limits of the physical properties that Si can withstand. Single crystal Silicon carbide (SiC) plays a significant role in the semiconductor industry due to its outstanding physical, chemical, and thermal stability [1,2]. However, SiC is also an archetypal hard and brittle semiconductor material that is hard to process because of its high hardness and brittleness. Dry etching and wet etching commonly used in semiconductor material processing are inefficient or even ineffective for micromachining of bulk SiC crystals [3,4]. Although some non-traditional machining forms, such as mechanical drilling and ultrasonic vibration, have also been applied in SiC micro-electrical devices, there are still problems of low integration efficiency and poor machining quality [5,6].

Recently, ultrafast lasers, generally referred to picosecond (ps) and femtosecond (fs) lasers, have been widely promoted as a crucial means for microfabrication of SiC devices, such as pressure sensors, acceleration sensors, actuators, power devices, and electronic skins, due to their unique advantages, including high processing efficiency, negligible collateral heat injury, and non-selectivity in material machining and cleanliness, and being non-polluting [7,8,9,10,11,12,13,14,15,16]. Although laser machining is not a parallel process similar to inductively coupled plasma etching (ICP) or reactive ion etching (RIE) for wafer-level processing, laser machining systems integrating precision CNC machines with high-speed scanning galvanometers have gradually made high-throughput fabrication of micro-nano structures on SiC substrate possible [17,18].

Although there have been numerous studies in the field of laser ablating of SiC for micro-nano structures, further research is necessary to provide a deeper, detailed insight and process optimization. For laser ablation of SiC, although the maximum energy absorption efficiency of infrared laser is lower than that of ultraviolet (UV) laser, the material removal process is faster [19]. Duc et al. measured the ablation threshold and the absorption coefficient of SiC through a single shot infrared (1064 nm) nanosecond pulse laser, and the values were 7.8 J/cm^2^ and 2.5 × 10^3^ cm^−1^, respectively [20]. Molian measured the ablation depth per pulse of 1–18 nm and the ablation threshold of 2 mJ/cm^2^ from a picosecond laser with a central wavelength of 1552 nm on 3C-SiC [21]. When the repetition rate of a laser pulse is less than 250 kHz, the laser-ablated trenches and holes indicated clean features and non-thermal damage. Zhang et al. studied the impacts of technological parameters on grooves of 4H-SiC processed by a femtosecond laser with a central wavelength of 800 nm and a maximum repetition rate of 1 kHz [22]. The depth and width of the grooves increased with increasing power, pulse repetition rate, and decreasing scanning speed. The surface roughness within 0.05 µm of the groove is obtained by increasing number of repeat scans. Wang et al. investigated the effect of repeated irradiation of femtosecond laser with a central wavelength of 1030 nm at near-threshold fluence of 1.1 J/cm^2^ on the surface morphologies induced in 4H-SiC surface [23]. The results show that the inoculation effect on the subsurface of 4H-SiC lead to different energy absorption accumulation, which was the reason for the discontinuity of the ablation zone. Iwatani et al. investigated laser drilling underwater on 4H-SiC by using a nanosecond pulsed infrared (1064 nm) laser [24]. When the laser fluence is less than 10 J/cm^2^ and the water thickness is 1 mm, vias without debris, heat-affected zones and cracks and were obtained. Wu et al. used a femtosecond laser with a central wavelength of 800 nm to ablate 4H-SiC in air, water, and hydrofluoric acid (HF), respectively [25]. The highest ablation threshold (4.98 J/cm^2^) occurred in air when the pulse number was 50 and the lowest ablation threshold (0.53 J/cm^2^) came from HF when the pulse number was 300. Li et al. studied the effects of processing parameters, including laser power, scanning speed, and scanning times, on the depth, width, and roughness (*Ra*) of microgrooves fabricated by a femtosecond laser with a central wavelength of 1064 nm on 4H-SiC through a response surface methodology and artificial neural network method [26].

In this paper, we study the ablation behavior of single crystal 4H-SiC when radiated by a femtosecond laser with a central wavelength of 1035 nm and a pulse width of 300 fs. An ablation threshold of a single-pulse and a multiple-pulse are measured, respectively. As the number of effective pulses increases, the cumulative effect causes the ablation threshold to drop and gradually reach a saturation value. Furthermore, the realization of square trenches with precise and controllable depth and low surface roughness are demonstrated to indicate the potentiality of infrared femtosecond laser micromachining in large-area and bulk ablation.

## 2. Materials and Methods

### 2.1. Materials

An n-type standard production grade 4H-SiC substrate doped with nitrogen from TankeBlue CO., Ltd., Beijing, China. with a thickness of 350 ± 25 μm, a resistivity of 0.015–0.025 Ω cm, and a surface roughness (*Sa*) in 640 × 640 μm^2^ regions of ≤100 nm is used in the experiments. The laser processing is carried out on the C surface of the substrate. The 4H-SiC sample is ultrasonically cleaned with acetone and absolute ethanol before and after laser ablation.

### 2.2. Laser Processing

**Figure 1:**
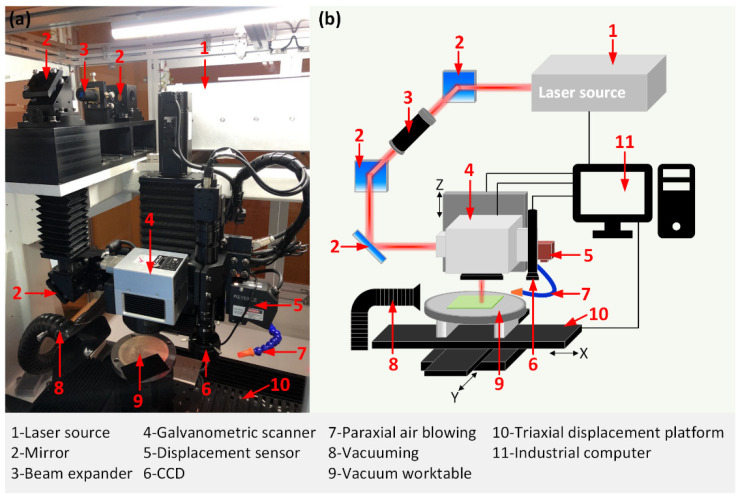
(**a**) Picture and (**b**) schematic of the laser workstation.

The experiment setup is shown in Figure 1. The infrared fiber laser (FemtoYL-40, YSL Photonics Co., Ltd., Wuhan, China) applied in this paper has the features shown in Table 1. The scanning traces of laser beam are controlled by a two-axis galvanometric scanner (SCANcube III 10, Scanlab, Inc., Puchheim, Germany) to realize fast positioning. An F-theta objective lens (RONAR SMITH SL-1064-70-100G, Wavelength Opto-Electronic (S) Pte. Ltd., Bukit Batok, Singapore) with an effective focal length of 100 mm enables precision focusing of the laser beam. During the experiment, the laser position is fixed and the positioning distance is monitored by a high-speed, high-accuracy CCD laser displacement sensor (KEYENCE, LK-G150).

## 3. Results and Discussion

### 3.1. Measurement of Single-Pulse Ablation Threshold

Much of the mechanism of the interaction between femtosecond lasers and materials is being explored, and the threshold theory has been widely recognized. The single-pulse energy of the laser beam follows a Gaussian distribution. The laser fluence *F*(*r*) can be expressed as:

(1) $$F(r)=F_{0}\exp(-2r^{2}/\omega_{0}^{2}),$$

where *F*_0_ is the peak energy density of the laser beam; *r* is the distance from any point on the spot to the center of the beam; *ω*_0_ is the beam waist radius of the Gaussian profile at the focus. The peak energy density *F*_0_ can be stated as follows:

(2) $$F_{0}=2P_{a}/(f\pi \omega_{0}^{2}),$$

where *P_a_* is the average laser power; *f* is the repetition rate of the laser. The laser ablation threshold and waist radius can be obtained through numerical fitting using Liu’s method as follows [27]:

(3) $$D^{2}=2\omega_{0}^{2}ln\left(F_{0}/F_{th}\right),$$

where *D* is the diameter of the laser ablation spot; *F_th_* is the ablation threshold. Substituting for the peak fluence from Equation (2):

(4) $$D^{2}=2\omega_{0}^{2}(lnP_{a}-ln\frac{\pi f\omega_{0}^{2}F_{th}}{2}),$$

**Figure 2:**
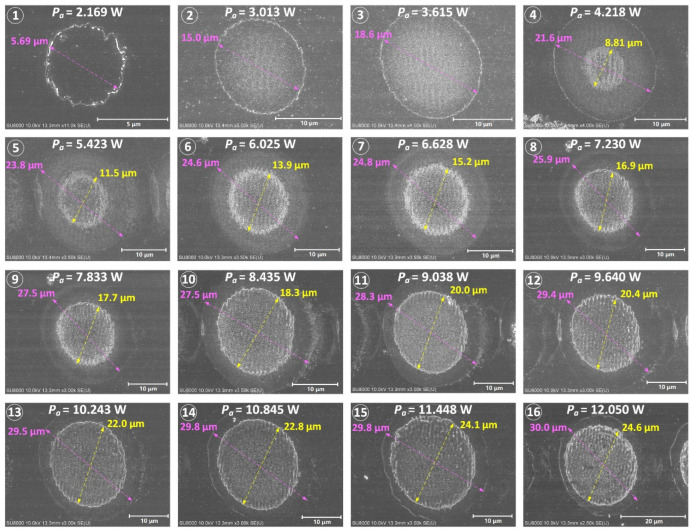
Microscopic view of ablation spots on the 4H-SiC under different average laser powers form 2.169 W to 12.050 W.

**Figure 3:**
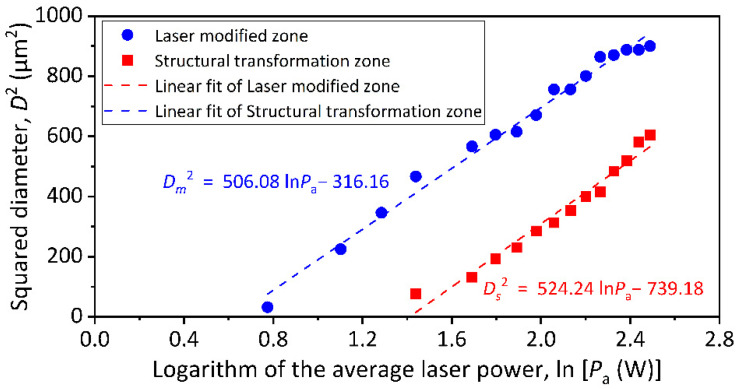
Fitting function curves of the squared diameters and the average laser powers.

In the actual femtosecond laser linear scanning process, the scanning speed of the laser focus is limited beyond the critical speed (this value is 6000 mm/s in the experiment) that enables pulse separation to obtain laser ablation spots of a single-pulse. The precise diameters of the laser ablation spots are obtained by microscopic measurement as illustrated in Figure 2. It can be found that when the average laser power reaches 4.218 W, the central region of the laser ablation spot separates a distinct sub-size spot. According to Shi [28], the region within the sub-size spot is the ablative phase transition zone and the annular region outside the sub-size spot is the material modified zone. According to Equation (4), the function curves corresponding to the square of laser ablation spot diameters (including the material modified spot diameter *D_m_* and the structural transformation spot diameter *D_s_*) and the logarithm of average laser powers are fitted in Figure 3. The beam waist radius *ω*_0_ can be calculated from the slope of the linear fitting line, and the estimated values are 15.91 μm and 16.19 μm, respectively. The single-pulse ablation thresholds for material modification (*F_th_m_*) and structural transformation (*F_th_s_*) are calculated from the intercepts with the abscissa axis as 2.35 J/cm^2^ and 4.97 J/cm^2^, respectively. Their corresponding laser average power are 1.868 W and 4.096 W, respectively.

### 3.2. Multiple-Pulse Scribing Ablation for Microgrooves

In the actual laser ablation process, the laser multiple-pulse machining is used instead of single-pulse machining. When the pulsed laser scans and scribes the surface of the material to be ablated, since the laser scanning speed cannot reach the speed of pulse separation, there will be an overlapping area of the light spot on the laser scanning track, and the same place will be affected by multiple-pulses. The combined action leads to the accumulation of laser pulses in the ablated area, forming heat accumulation, that is, the laser pulse energy accumulation effect. The effective pulse number is defined as the average accumulated pulse number in the processing area to quantify the number of laser pulses under the pulse accumulation effect more specifically [29]:

(5) $$N=K\frac{2\omega_{0}f}{v},$$

where *K* is the number of laser scans; v is the laser scanning speed.

**Figure 4:**
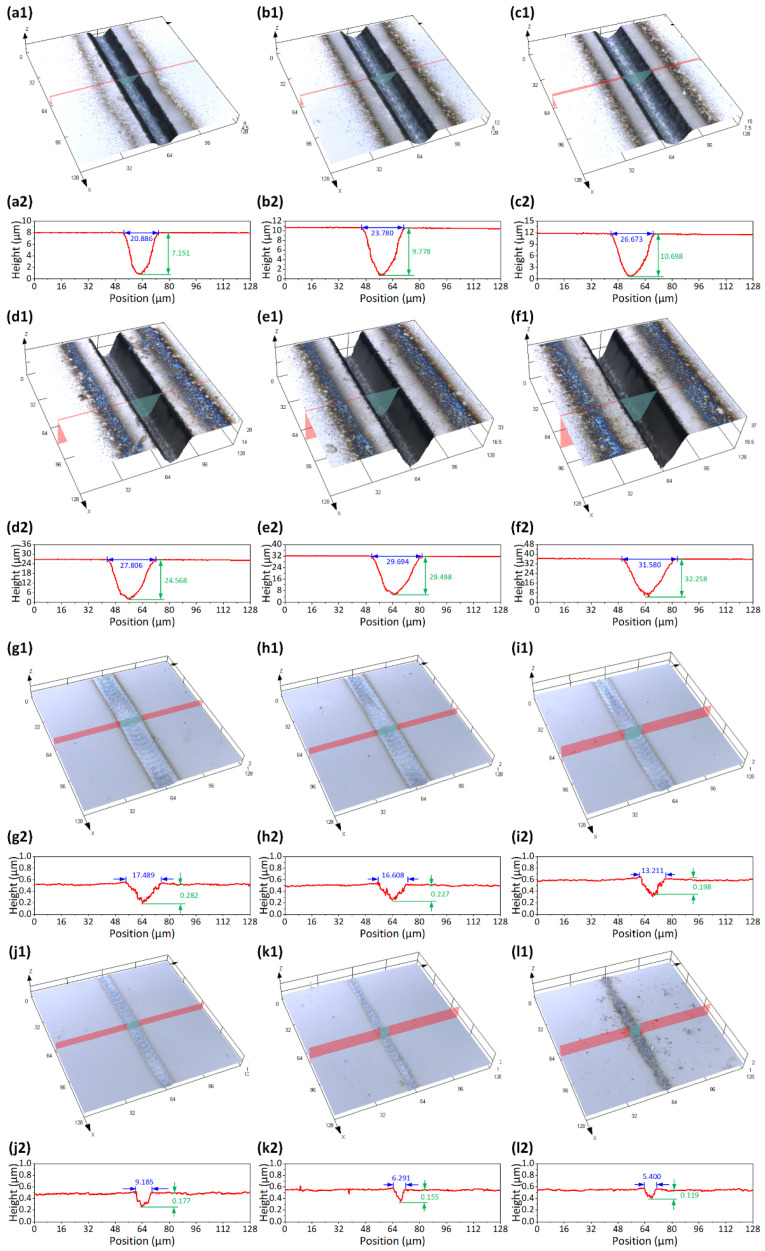
LSCM images of ablated microgrooves (*N* = 240) at different average laser powers of (**a1**) 1.687 W, (**b1**) 1.928 W, (**c1**) 2.169 W, (**d1**) 2.410 W, (**e1**) 2.651 W, and (**f1**) 2.892 W and their profile data in (**a2**–**f2**). LSCM images of ablated microgrooves (*N* = 12) at different average laser powers of (**g1**) 1.687 W, (**h1**) 1.928 W, (**i1**) 2.169 W, (**j1**) 2.410 W, (**k1**) 2.651 W, and (**l1**) 2.892 W and their profile data in (**g2**–**l2**).

In this experiment, different average laser powers of 1.687 W, 1.928 W, 2.169 W, 2.410 W, 2.651 W, and 2.892 W are set for scribing ablation of 4H-SiC. The laser repetition frequency (*f*) is fixed at 200 kHz, and the scanning speed (*v*) is fixed at 1000 mm/s. The laser scanning times (*K*) are set to 2, 5, 10, 20, 20, 40, 80, 120, 160, 200, 240, and 280, respectively, and the corresponding effective pulses (*N*) are 12, 30, 60, 120, 240, 480, 720, 960, 1200, 1440, and 1680, respectively. Hence, there are a total of 66 ablated microgrooves at different effective pulse numbers. On account of the large number of total experimental groups, only the images of ablated microgrooves processed by the effective pulse number of 240 are shown in Figure 4(a1–f1). Their profile data in Figure 4(a2–f2).

**Figure 5:**
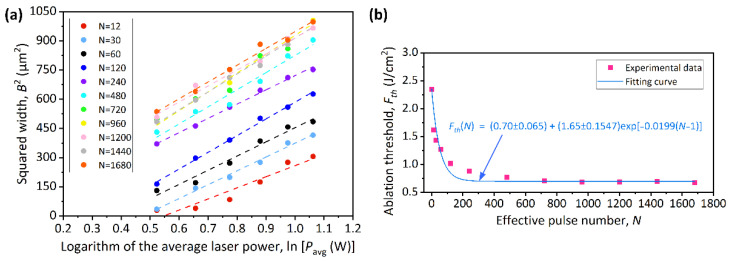
(**a**) The widths of all the ablated microgrooves versus the average laser powers under different effective pulse numbers. (**b**) The relationship between effective pulse number and ablation threshold.

The fitting curves of the relationships between the widths (*B*) of ablated microgrooves and the average laser powers (*P_a_*) under different effective pulse numbers are described in Figure 5a. According to the method in Section 3.1, the multiple-pulse ablation threshold versus different effective pulse numbers is illustrated in Figure 5b. The fitting curve of the experimental data of multiple-pulse ablation follows the laser pulse energy accumulation model. It can be expressed as [30]:

(6) $$F_{th}(N)=F_{th}(\infty )+\left[F_{th}\left(1\right)-F_{th}(\infty )\right]exp\left[-k(N-1)\right],$$

where *F_th_*(*N*) represents the corresponding ablation threshold when the effective pulse number is *N*; *F_th_*(*∞*) is the saturated ablation threshold when the effective pulse number is infinite; *F_th_*(1) is the single-pulse ablation threshold; *k* is an incubation parameter (independent of *N* in first approximation) that describes the intensity of defect accumulation and the enhancement in photon absorption. The larger *k* is the fewer effective pulse number *N* are needed to obtain the constant saturated ablation threshold *F_th_*(∞), below which an infinite number of laser pulses would not damage the structure of 4H-SiC. It can be found that when the *N* is less than 240, the multiple-pulse ablation threshold decreases rapidly with the raising of the *N*. When the *N* reaches 720, the laser pulse energy accumulation effect reaches saturation and the ablation threshold is 0.70 J/cm^2^, which is a 70% reduction compared to the single-pulse ablation threshold for material modification (*F_th_m_*). When the ablation threshold is saturated, it can be considered that *F_th_*(*∞*) = *F_th_*(720) = 0.70 J/cm^2^. According to the experimental result of single-pulse ablation in Section 3.1, *F_th_*(1) = *F_th_m_* = 2.35 J/cm^2^. Thus, the calculated incubation parameter *k* is 0.0199. It is worth noting that the saturated ablation threshold *F_th_*(*∞*) measured by multiple-pulse experiment is less than the single-pulse ablation threshold for material modification (*F_th_m_*). This means that even if the laser fluence is lower than *F_th_m_*, as long as it is higher than the saturated ablation threshold *F_th_*(*∞*), the subsequent macroscopic damage of the material can be promoted when the *N* accumulates to a certain value. This conclusion can also be obtained from the ablation experiments of other materials [31].

**Figure 6:**
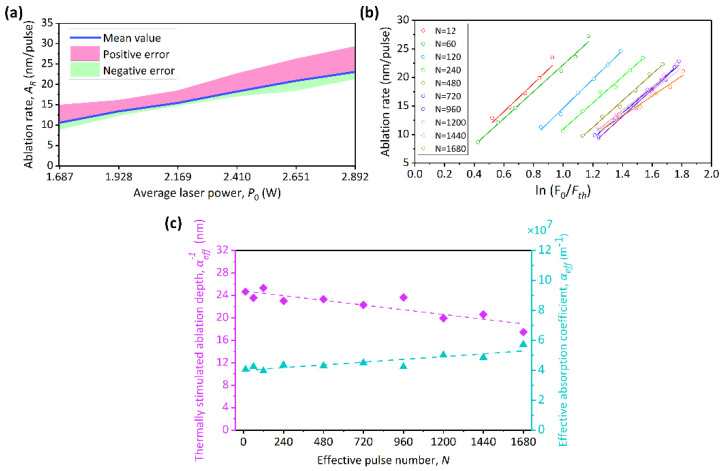
(**a**) The ablation rate of the microgrooves versus the average laser power. (**b**) The ablation rate versus ln(*F_0_*/*F_th_*) for different effective pulse numbers. (**c**) Results of the thermally stimulated ablation depth and the effective absorption coefficient for different effective pulse numbers.

A controllable and accurate ablation rate is very important for micro/nano machining. The laser ablation depth of per effective pulse is defined as the ablation rate (*A_R_*), which is estimated through dividing the depth of the ablated microgroove by the effective pulse number [32]:

(7) $$A_{R}{=\alpha }_{\mathrm{eff}}^{-1}\cdot \ln\left(F_{0}/F_{\mathrm{th}}\right),$$

where *α_eff_* is the effective absorption coefficient. Its reciprocal is defined as the thermally stimulated ablation depth, which includes the thermal diffusion length and the light penetration depth [21]:

(8) $$\alpha_{\mathrm{eff}}^{-1}=\alpha^{-1}+2{\left(\kappa \tau \right)}^{1/2},$$

where *α* is the linear absorption coefficient, *κ* is the thermal diffusivity, and *τ* is the pulse width. In the case, the energy can be transmitted in the subsurface region optically (via absorption) and thermally (via heat diffusion). These processes are simultaneous. The depths (*H*) of 66 microgrooves are also recorded, some of which are shown in Figure 4. The ablation rate of the microgrooves exhibits an evident linear relationship with the average laser power, as depicted in Figure 6a. The error band represents the deviation of the ablation rate under different effective pulse numbers. This means that the ablation rate can be effectively controlled by the average laser power. Furthermore, the ablation rate versus ln(*F*_0_/*F_th_*) for different effective pulse numbers is plotted in Figure 6b. According to Equation (7), the slope of each linear fitting function in Figure 6b represents the thermally stimulated ablation depth of 4H-SiC. The effective absorption coefficient *α_eff_* of 4H-SiC can be furtherly calculated. The relationship between the thermally stimulated ablation depth and the effective absorption coefficient and the effective pulse number is shown in Figure 6c. The arithmetical average of the thermally stimulated ablation depth of 4H-SiC is 22.4 nm, which is comparable to the result of (21 nm) [33]. There is evidence that the surface temperature of the material in the laser irradiation region would rise with the increase in the pulse number [34]. The *α* would increase with rising temperature due to the ionization of the dopant and thermal activation of the valence band electrons [35,36].

### 3.3. Large-Area and Bulk Ablation

#### 3.3.1. Analysis of Process Parameters Effect on the Material Removal Rate

The use of lasers for large-area bulk ablation of 4H-SiC is further investigated. According to Equation (5), the average laser power (*P_a_*) is fixed at 2.169 W, and the repetition frequency (*f*) is fixed at 200 kHz, so different effective pulse numbers (*N*) are obtained by changing the scanning speed (*v*) and the number of scans (*K*) to process square trenches with dimensions of 1 × 1 mm^2^. The hatch style of laser scanning is to fill a series of parallel paths. The effect of hatch spacing is also considered. The parameter combinations of the experimental groups are shown in Table 2.

**Figure 7:**
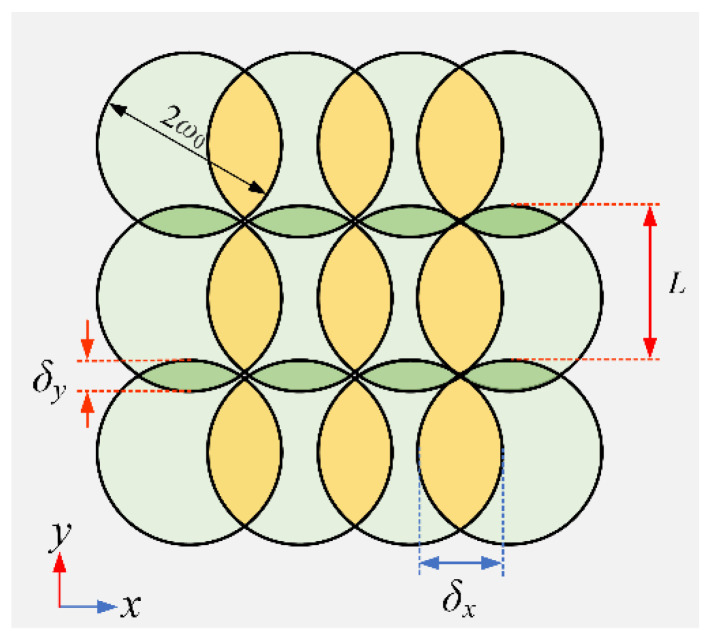
Illustration of overlapping ratios of the laser spot in *x* (*δ_x_*) and *y* (*δ_y_*) directions.

The overlapping ratios of the laser spot in *x* (*δ_x_*) and *y* (*δ_y_*) directions, as shown in Figure 7, are precisely controlled respectively through setting laser scanning speed (*v*) and hatch spacing (*L*) [37]:

(9) $$\delta_{x}=\left(1-\frac{v}{2w_{0}f}\right)\times 100\%,$$

(10) $$\delta_{y}=\left(1-\frac{L}{2w_{0}}\right)\times 100\%,$$

Table 3 shows the overlapping ratios corresponding to different laser scanning speed (*v*) and hatch spacing (*L*) obtained according to Equation (10) under the premise of constant laser spot size and repetition frequency.

**Figure 8:**
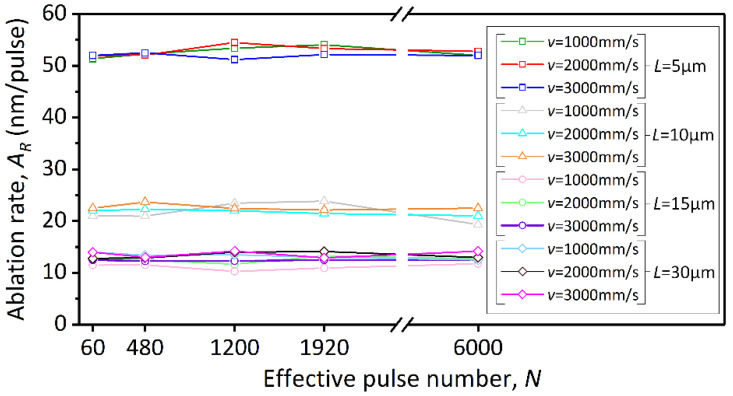
The ablation rate (*A_R_*) of the trenches versus the effective pulse number (*N*) at different hatch spacings (*L*).

**Figure 9:**
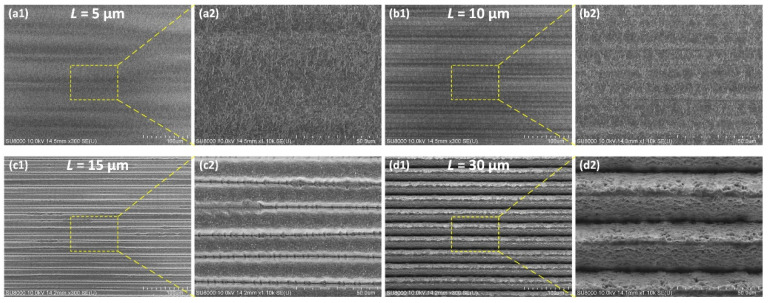
The bottom surface topography of deep trenches machined using different hatch spacings of (**a1**) 5 μm, (**b1**) 10 μm, (**c1**) 15 μm and (**d1**) 30 μm and their enlarged views of (**a2**–**d2**) at the average laser power of 2.169 W and the scanning speed of 1000 mm/s.

As shown in Figure 8, at the same hatch spacing (*L*), the ablation rate (*A_R_*) is almost constant even if the effective pulse number (*N*) varies. This is important for the machining requirements of large-area bulk ablation, and it means that a precise amount of material removal can be effectively controlled to achieve the desired depth of structure. Moreover, the ablation rate (*A_R_*) defined by the effective pulse number (*N*) is not affected by the scanning speed at the same hatch spacing (*L*). The effect of hatch spacing (*L*) on laser ablation behavior is significant. Figure 9 illustrates the bottom surface topography of deep trenches machined using different hatch spacings. As the hatch spacing increases from 5 μm to 15 μm, *δ_y_* decreases, that is, the effective ablation of the material in the overlapping area of the laser spot in the y direction weakens, and the ablation rate (*A_R_*) decreases. When the hatch spacing reaches 30 μm, *δ_y_* is 0%, and the microgrooves scribed by each scanning path are separated from each other, resulting in an ablation rate (*A_R_*) substantially equal to that of scribing ablation shown in Figure 6a under the same average laser power. Essentially, the ablation rate hardly decreases after *δ_y_* is below 50%.

**Figure 10:**
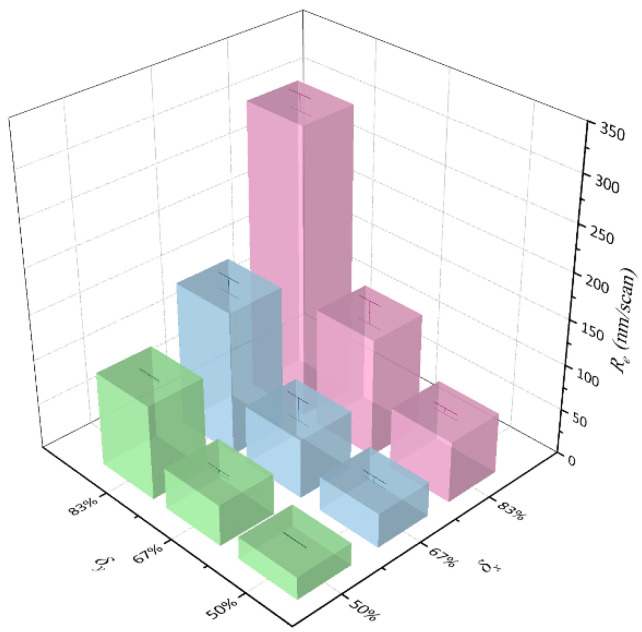
The material removal rate (*R_e_*) versus the overlapping ratios of the laser spot in *x* (*δ_x_*) and *y* (*δ_y_*) directions at the average laser power of 2.169 W.

In fact, in the process of deep-structure processing with laser scanning layer-by-layer, the depth of material removed per scan is usually defined as the material removal rate (*R_e_*):

(11) $$R_{e}=H/K,$$

where *H* is the machining depth of material; *K* is the number of laser scans. Thus, the effect of scanning speed will be taken into account. As plotted in Figure 10, we converted the ablation rate (*A_R_*) for *L* = 5, 10 and 15 μm in Figure 8 to the material removal rate (*R_e_*), and converted *v* and *L* to *δ_x_* and *δ_y_*, respectively. Error bars represent the deviation in calculated *R_e_* when machining the trenches through different times of scans. It can be found that the decrease of *δ_x_* and *δ_y_* will lead to the reduction of *R_e_*. When *δ_x_* and *δ_y_* decrease by the same amount, the contribution of *δ_y_* to the reduction of *R_e_* is greater than *δ_x_*.

**Figure 11:**
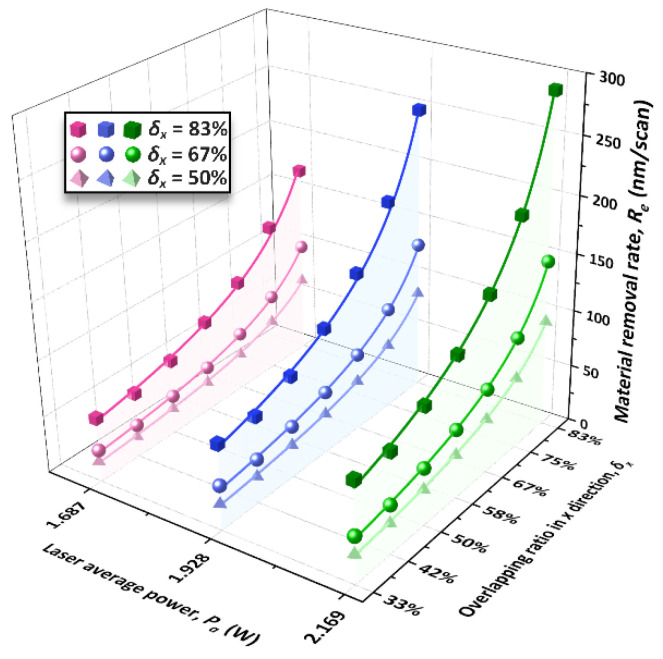
The material removal rate (*R_e_*) versus the overlapping ratios of the laser spot at different average laser powers.

Moreover, the effects of *δ_x_* and *δ_y_* on the *R_e_* at different *P_a_* of 1.687 W, 1.928 W, and 2.169 W are graphed together in Figure 11. It can be found that at different *P_a_* and *δ_y_*, *R_e_* drops gradually with the decrease of *δ_x_*. When *δ_x_* is lower than 50%, that is, the scanning speed exceeds 3000 mm/s, the weakening effect of *δ_x_* on *R_e_* is no longer obvious. The material removal behavior reaches an approximate saturation rate.

#### 3.3.2. Planarization Processing Surface

**Figure 12:**
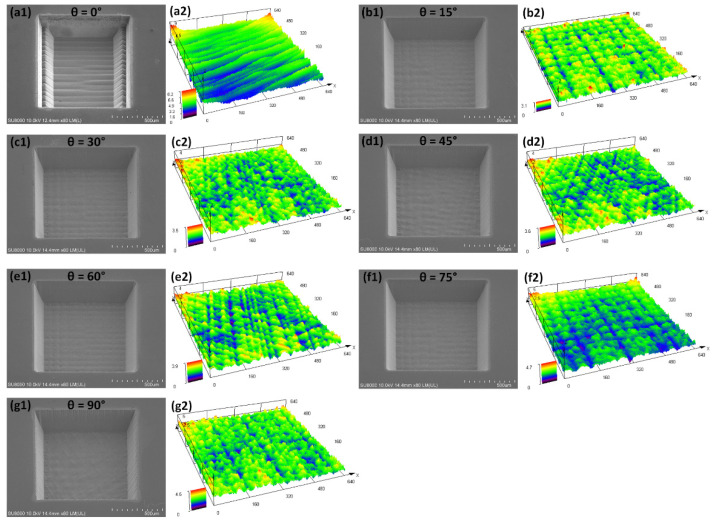
(**a1**–**g1**) SEM images and (**a2**–**g2**) LSCM measurements of the square trenches machined only by changing the hatch rotation angle (*θ*) from 0° to 90° at the laser average power of 2.169 W, the scanning speed of 1000 mm/s, and the hatch spacings of 5 μm.

A certain hatch rotation angle of laser scanning is set between each scan covering the machined-area to prevent the laser from repeatedly ablating the same (*x*, *y*) coordinate point in the procedure of multi-layer scanning. Figure 12 shows the square trenches machined only by changing the hatch rotation angle (*θ*). Surface roughness (*Sa*) is measured from an area in the range of 640 × 640 μm. When *θ* = 0°, the square trench has different depths at both ends of the hatch line due to the influence of the laser delay. The bottom surface of the trench presents a distinct furrow shape. When the *θ* = 15°, 30°, 60°, and 75°, both the bottom and sidewalls of the square trenches appear to be extremely smooth with inconspicuous melt formation, microcracks, or other thermal damages. When the *θ* = 45° and 90°, the sidewall of the square trenches exhibits wrinkles at the outlet position.

**Figure 13:**
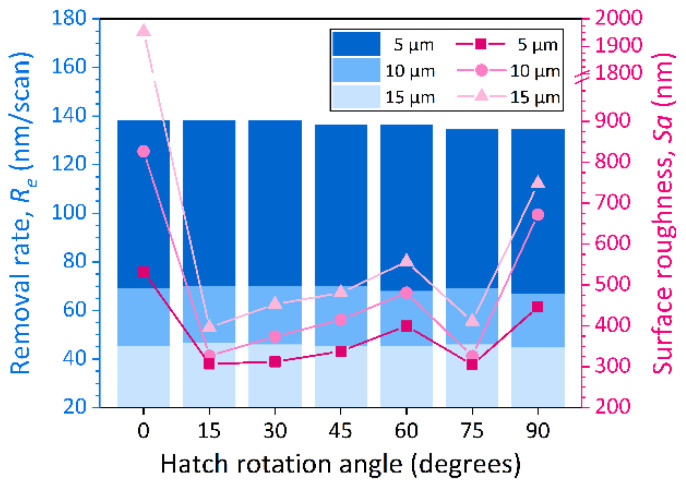
The material removal rate (*R_e_*) and surface roughness (*Sa*) versus the hatch rotation angles at different hatch spacings of 5, 10, and 15 μm.

Further results in Figure 13 illustrate that the changes of the bottom surface roughness of the square trenches machined with different hatch spacings show consistent regularity. The *Sa* is minimized at hatch rotation angles of 15° and 75° at different hatch spacings. Lower *Sa* is obtained by a smaller hatch spacing of 5 μm. In addition, Figure 13 also shows that the material removal rate (*R_e_*) is insensitive to changes in hatch rotation angle. This means that the precise amount of material removal can still be controlled while modulating the flatness of the ablated features. 

## 4. Conclusions

In this paper, a fundamental study about the ablation behavior of 4H-SiC single crystal by infrared femtosecond laser is presented. The single-pulse ablation thresholds for material modification and structural transformation are 2.35 J/cm^2^ and 4.97 J/cm^2^, respectively. When the effective pulse number reaches 720, the ablation threshold of 4H-SiC would drop to 0.70 J/cm^2^ due to the laser pulse energy accumulation effect. The calculated average of the thermally stimulated ablation depth of 4H-SiC is 22.4 nm. The phenomenon that the absorption coefficient gradually decreases with the increase of the effective pulse number proves that heat accumulation still exists during the multiple-pulse ablation process. In large-area bulk ablation processing, the ablation rate per pulse is almost constant even if the effective pulse number varies. The reduction of laser spot overlapping ratio in *x* direction has a greater weakening effect on the material removal rate than that in *y* direction. The surface roughness is minimized at hatch rotation angles of 15° and 75° at different hatch spacings of 5, 10, and 15 μm. Changes in the hatch rotation angle do not result in variations in material removal rates.

## Figures and Tables

**Table 1:** Characteristic parameters of laser source.

Model	Value
Central wavelength	1035 nm
Pulse width	300 fs
Maximum output power	40 W
Repetition rate	25 kHz–5 MHz
Power stability	<2%
Pulse energy	80 μJ
Beam quality	M2 < 1.3
Beam divergence	<2 mrad

**Table 2:** The laser parameter combinations of the experimental groups.

Number	Scanning Speed (mm/s)	Number of Scans	Effective Pulse Number	Hatch Spacing (μm)
1	1000	10	60	5, 10, 15, 30
2	2000	20		
3	3000	30		
4	1000	80	480	5, 10, 15, 30
5	2000	160		
6	3000	240		
7	1000	200	1200	5, 10, 15, 30
8	2000	400		
9	3000	600		
10	1000	320	1920	5, 10, 15, 30
11	2000	640		
12	3000	960		
13	1000	1000	6000	5, 10, 15, 30
14	2000	2000		
15	3000	3000		

**Table 3:** Characteristic parameters of laser source.

*v* (mm/s)	Δ*x*	*L* (μm)	*δy*
1000	83%	5	83%
2000	67%	10	67%
3000	50%	15	50%
6000	0%	30	0%
